# Discovering the Impact of Preceding Units' Characteristics on the Wait Time of Cardiac Surgery Unit from Statistic Data

**DOI:** 10.1371/journal.pone.0021959

**Published:** 2011-07-19

**Authors:** Jiming Liu, Li Tao, Bo Xiao

**Affiliations:** Computer Science Department, Hong Kong Baptist University, Hong Kong, Special Administrative Region, People's Republic of China; University of Giessen Lung Center, Germany

## Abstract

**Introduction:**

Prior research shows that clinical *demand* and supplier *capacity* significantly affect the *throughput* and the *wait time* within an isolated unit. However, it is doubtful whether characteristics (i.e., *demand*, *capacity*, *throughput*, and *wait time*) of one unit would affect the *wait time* of subsequent units on the patient flow process. Focusing on cardiac care, this paper aims to examine the impact of characteristics of the catheterization unit (CU) on the *wait time* of cardiac surgery unit (SU).

**Methods:**

This study integrates published data from several sources on characteristics of the CU and SU units in 11 hospitals in Ontario, Canada between 2005 and 2008. It proposes a two-layer wait time model (with each layer representing one unit) to examine the impact of CU's characteristics on the *wait time* of SU and test the hypotheses using the Partial Least Squares-based Structural Equation Modeling analysis tool.

**Results:**

Results show that: (i) *wait time* of CU has a direct positive impact on *wait time* of SU (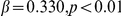); (ii) *capacity* of CU has a direct positive impact on *demand* of SU (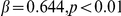); (iii) within each unit, there exist significant relationships among different characteristics (except for the effect of *throughput* on *wait time* in SU).

**Conclusion:**

Characteristics of CU have direct and indirect impacts on *wait time* of SU. Specifically, *demand* and *wait time* of preceding unit are good predictors for *wait time* of subsequent units. This suggests that considering such cross-unit effects is necessary when alleviating *wait time* in a health care system. Further, different patient *risk* profiles may affect *wait time* in different ways (e.g., positive or negative effects) within SU. This implies that the wait time management should carefully consider the relationship between priority triage and risk stratification, especially for cardiac surgery.

## Introduction

The impact of highly fluctuating *demand* (patient inflow) and available service *capacity* on the *performance* of a health care system deserves long standing attention [1] [2]. As a key characteristic of a health care system, *demand* is often represented by the number of visits to services [3] [4] or the expenditures on services [5] [6]. There are many factors affecting the demand of a health care system, including increasing number of patients due to the aging and rising population [7], the growing incidence of diseases such as diabetes [8], the development of diagnostic and treatment technology [7], patient status such as the seriousness of the illness [9], the position of the patient on a waiting list [10], the geographic distance to the services [11], patient personal profile (e.g., demographics [12], socioeconomic condition [13] [14]), and unpredictable patient behaviors like balking, reneging, jockeying, and repeating [15] [16] [17] [18].

Another important characteristic of a health care system is *capacity*, which denotes the resources (e.g., financial, human, physical) available to meet the demand [19] [20]. *Capacity* is usually judged by the quantity and quality of resources at hand [7] [21] or the working time available [22]. Commonly interested factors affecting the capacity include human resources such as skilled doctors and assistants (e.g., nurses, anesthetists) [21], physical resources such as beds and equipments [7], management strategies such as resources utilization and allocation [23], resource planning and scheduling [23] [24].

The third important characteristic of a health care system is *performance*. Two common indicators of *performance* are *throughput* and *wait time* [15] [25] [26]. *Throughput* is typically quantified by counting the number of patients who have received a needed health care service in a given time period [27]. It is thus a way to observe the utilization of resource. Different from *throughput*, *wait time* is the amount of time a patient has to wait for receiving a needed health care service [25] [28]. Wait time is a particular concern in health care, especially for such key services as catheterization and cardiac surgery. Long wait time is not only an impediment to quality care but also a risk factor for patients [29] [30]. There are various measurements for wait time, such as median wait time (i.e., the point at which half of the patients have received their treatment with the other half still waiting), and queue length (i.e., the total number of patients in the waiting list) [25] [28]. *Wait time* is often different depending on patient urgency categories. In a government dominated health care system (e.g., Hong Kong, or Canada), each patient who waits in the key units is assigned an urgency rating score according to the presenting symptoms [31] [32] [33]. Wait time strategies are adopted based on different urgency categories [25]. The higher urgent score a patient has, the shorter time s/he will wait.

Prior research has investigated the relationships among *demand*, *capacity*, *throughput*, and *wait time* empirically for a long time. It has revealed that *demand* has a significant impact on *capacity* [34], *throughput*, and *wait time* in various units (e.g., congested recovery room, emergency department) [21] [35] [36] [37]. *Capacity* has also been found to exert a significant negative influence on *throughput* and *wait time* [21] [35] [36] [37] [38]. Although some researchers argue that *capacity* has a positive impact on *demand* (higher capacity attracting more patients coming to hospitals, especially the non-urgent patients) [39] [40], such argument has not been supported with plenty empirical evidence [41]. In addition, although prior research suggests that the improvement of throughput often accompanies the reduction of wait time [42], the impact of *throughput* on *wait time* has not been empirically investigated.

**Figure 1:**
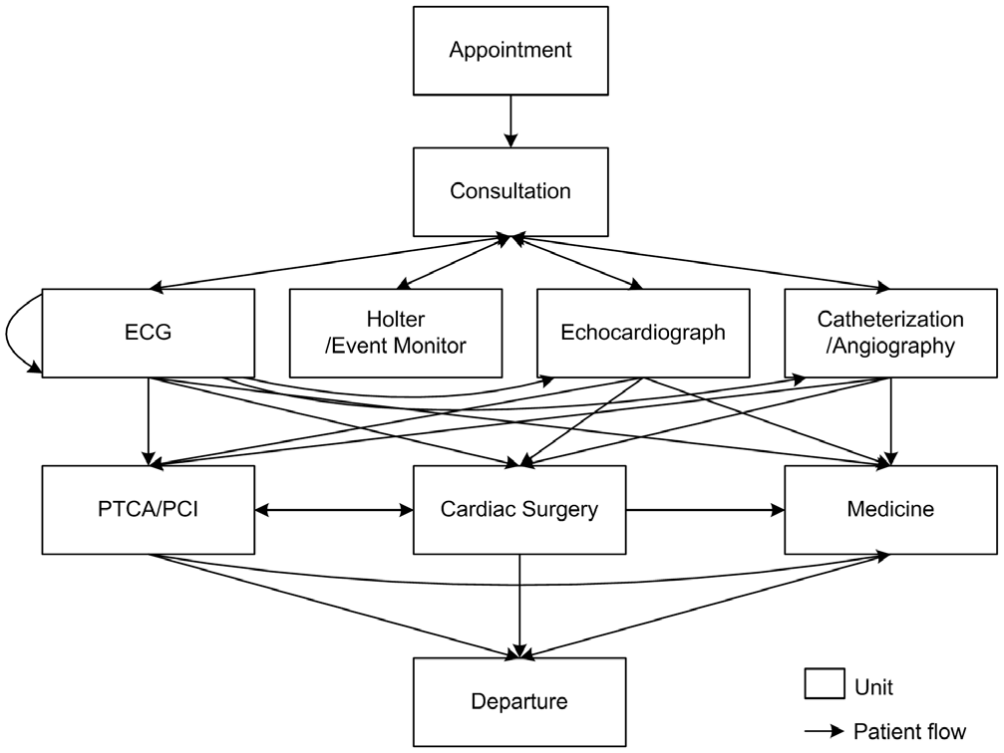
The unit framework of cardiac care drawn from the cardiac treatment guidelines [**45**] [**46**]. (ECG: Electrocardiogram; PTCA: Percutaneous transluminal coronary angioplasty; PCI: Percutaneous coronary intervention.)

Health care units and services have generally evolved in silos focusing on satisfying their own customers [43]. Accordingly, extant research has focused on the relationships among the characteristics within a specific unit. However, we argue that it is inadequate to examine the within-unit relationships in isolation [43] [44], because, in the real world, all the units in a health care system are networked via patient flow. For example, based on the cardiac treatment guidelines [45] [46], units involved in the cardiac care are sequentially connected according to patient visits (Figure 1). Two units with a directed link denote they are temporally related, i.e., patients usually visit the unit the arrow points toward (i.e., subsequent unit) after visiting the unit the arrow points away from (i.e., preceding unit). There usually exits a “funnel and filter” effect [47], p.163 (i.e. preceding units determine the actual numbers and the throughput for patients proceeding into the subsequent units) between two temporarily related units. In the context of the catheterization unit (CU) and the cardiac surgery unit (SU), a “diagnostic-therapeutic” cascade effect [48, p.2797] (more catheterization diagnostic tests performed are also likely to have more cardiac surgeries) may also exist [49] [50]. Thus, investigating the impact of the cross-unit relationships, in addition to within-unit relationships, may reveal more important insights for wait time management [44].

**Figure 2:**
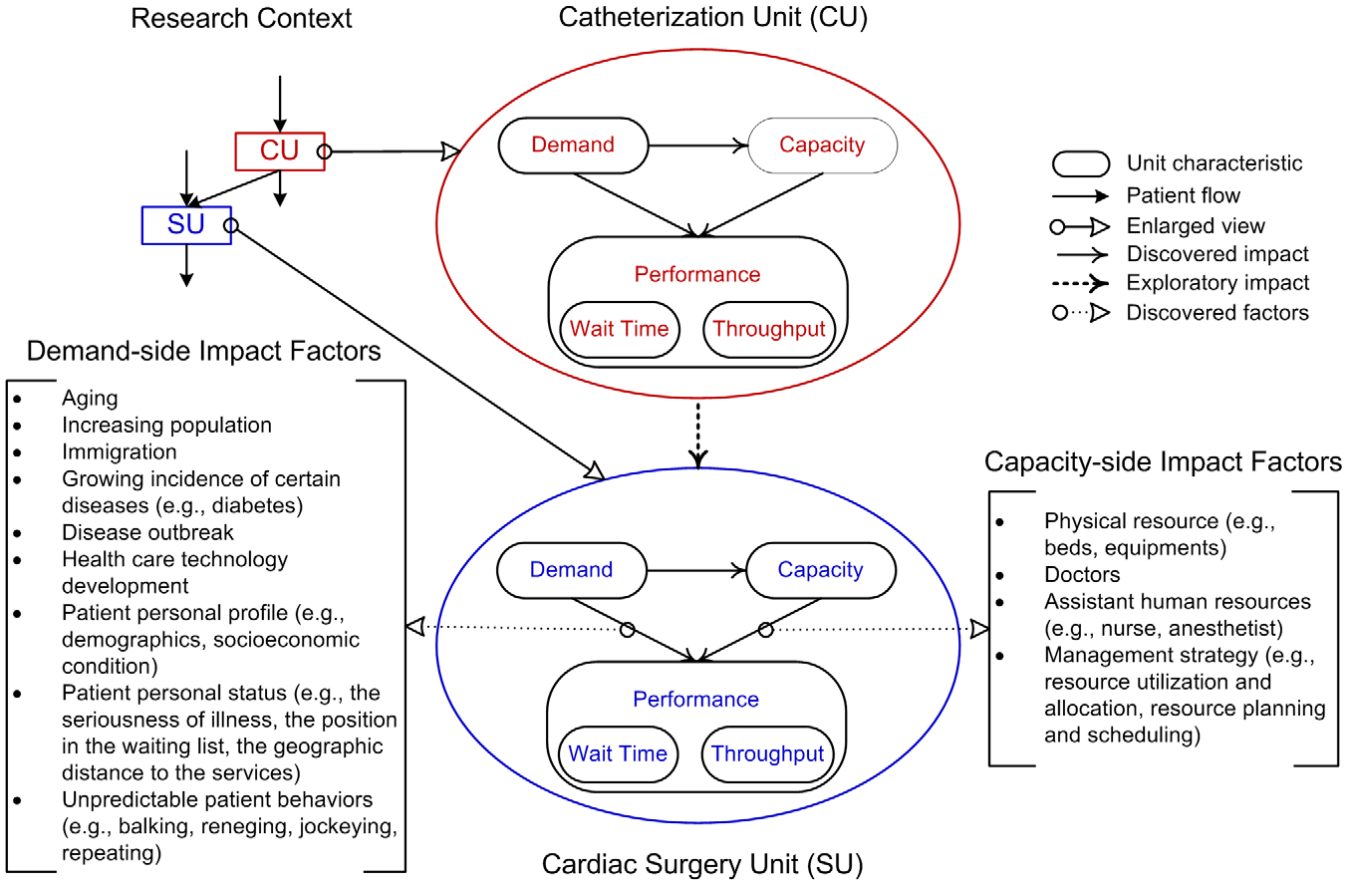
The research framework with the summarization of the impact factors for throughput and wait time.

In sum, the impact factors for a health care unit's performance (i.e., *wait time*, and *throughput*) have been studied from the demand-side and capacity-side perspectives (shown in Figure 2). The relationships among *demand*, *capacity*, *throughput*, and *wait time* have been investigated within a unit. However, little attention has been paid to the relationships among the characteristics in a cross-unit context, a gap this study aims to fill. In this study, we explore whether and how the characteristics of one unit exert an influence on the characteristics (*wait time* in particular) of other temporally related units (Figure 2 shows the overall research framework). We choose the CU and the SU as our research context, because (i) they both provide key services [25] [28], (ii) they are temporally connected [51], and (iii) published data about the two units are available (http://www.ccn.on.ca/). We propose a two-layer wait time model (see detailed discussion in the next section) to investigate the CU's characteristics on the wait time of SU, with each layer representing a unit. Both within-unit and cross-unit relationships are represented in the model.

We employ the Structural Equation Modeling (SEM) [52] [53] to explore the underlying relationships among the characteristics of two units (i.e., CU and SU). Compared to traditional statistic techniques (e.g., regression, ANOVA), the SEM (i) has the ability to construct latent variables (abstract concepts cannot be measured directly) [54], and (ii) permits exploring and confirming complex (e.g., hierarchical or non-hierarchical, recursive or non-recursive) variable relationships concurrently, in addition to traditional pairwise variable relationships [54] [55]. As a result, the SEM enables us to identify the complete causal paths of the cross-unit relationships among latent variables (i.e., *demand*, *capacity*, *throughput*, and *wait time* in this study), which are not supported by any traditional statistic method individually.

The data for this study is obtained from the Cardiac Care Network of Ontario and the Ontario Physician Human Resources Data Centre. We choose such data because it has been collected and released by the Ontario government regularly for more than ten years. It provides comprehensive information on health care services in Ontario for carrying out our research.

## Methods

### Hypotheses and Research Model

It has been recognized that matching the fluctuating demand for health care systems with the capacity available is vital for bettering the outcomes (e.g., morbidity and mortality rate, or wait time) [56]. Thus, there has been extensive research examining the relationships among *demand*, *capacity*, *throughput*, and *wait time*, especially within a single unit.

Prior research has shown that *demand* has a positive impact on *throughput* and *wait time*. For example, Asaro et al. [37] found in the context of an emergency department that increasing the arrivals (i.e., demand) increased the throughput and the wait time. Harindra et al. [21] showed that clinical demand was an important factor for the access inequalities (i.e., wait time) of catheterization in Canada. Schoenmeyr et al. [35] revealed a sensitive relationship between the caseload (i.e., demand) and the wait time in a congested recovery room. Harewood et al. [36] found that annual wait time for routine endoscopic procedures lengthened dramatically because of a significant increase in annual procedure demand on endoscopy services. Therefore, we hypothesize that *demand* has a positive impact on *throughput* (**Hypothesis 1**, **H1**) and *wait time* (**Hypothesis 2**, **H2**).

In analyzing the current research on the relationship between *demand* and *capacity*, Baker [34] noted that the desire to meet patient demands was a dominant driving force for capacity changing. Buerhaus [57] pointed out that demand increasing for aging population may result in expanding nursing workforce (human resources) to avoid threatening the health care quality. Justman et al. [58] indicated that HIV scale-up needed to develop laboratory systems and infrastructures (i.e., physical resources). Several researchers have argued that *capacity* has a positive impact on *demand* [39] [40]. For instance, Smethurst and Williams [39] [40] noted that for each specific disease, there were many more patients who did not visit the doctors than those who did visit (i.e., “hidden” patients [39, p.653]). To meet these potential overwhelming demand, the supplier may increase the capacity. Changes in the capacity may trigger changes in demand because more patients are then attracted to the service providers. However, this argument has not been evidently tested [41]. Therefore, in this study, we hypothesize that *demand* has a positive impact on *capacity* (**Hypothesis 3**, **H3**), and *capacity* does not have an effect on *demand*.

Regarding the impact of *capacity* on *throughput* and *wait time*, prior research has indicated that *capacity* is important to ensure better performance (e.g., *throughput*, *wait time*) of a health care system. For instance, Harindra et al. [21] found that supplier capacity was an important factor determining access inequalities (i.e., wait time) of catheterization in Canada. Schoenmeyr et al. [35] showed that the physical capacity of supplier (e.g., beds) had a significant impact on the wait time in a congested recovery room. Trzeciak and Rivers [38] also found that inpatient capacity (e.g., beds) had an effect on the throughput in an emergency department. Harewood et al. [36] further showed that modifications in routine clinical practice (i.e., service capacity) could significantly affect a procedure's wait time.

A few studies have revealed that improving the *capacity* may help improve the *throughput* and the *wait time* of a health care unit. Mukherjee [59] found that improving the management of physicians (e.g., staffing mix) improved patient throughput. Others showed that improving the capacity management (such as employing intelligent patient scheduling) shortened the wait time efficiently [60]–[61]. Therefore, in this study, we hypothesize that *capacity* has a positive impact on *throughput* (**Hypothesis 4**, **H4**) and *wait time* (**Hypothesis 5**, **H5**) within a unit.

Little prior research has investigated the relationship between *throughput* and *wait time*. Brenner et al. suggested that the improvement of throughput often accompanied the reduction of wait time [42]. An intuitive explanation is that given a stable demand (i.e., determined number of arrivals) in a unit, if resources (physical or human resources) in this unit can be more efficiently used, the patients may be treated quicker. So that the wait time of each patient may be shortened. Therefore, in this study, we hypothesize that *throughput* has a negative impact on *wait time* (**Hypothesis 6**, **H6**) within a unit.

Prior research has examined the relationships of characteristics among several units within a hospital. Reported by Alter et al. [47 p.163], the catheterization has a “funnel and filter” effect on the cardiac surgery. That means the demand and the capacity of CU determine the actual numbers and the throughput for patients proceeding into the SU. Similarly, prior research has revealed that the CU and the SU have a “diagnostic and therapeutic” cascade effect [48, p.2797] [49] [50]. This implies that more catheterization diagnostic tests performed in CU may trigger more patients to undergo cardiac surgeries. Some studies have examined the interrelationships among different units within a hospital for bed allocation [62] [63] [64]. Results showed that bed allocations for patients were influenced by the capacities of all the units. However, such research does not explain clearly how and to what extent the capacity of one unit may influence the wait time of another. In addition, to the best of our knowledge, no prior research has studied whether and to what extent the wait time of one unit influences the wait time of a temporally related unit. In this study, we explore such a wait time relationship between the CU and the SU and hypothesize that (i) *demand* of CU has a positive impact on *demand* of SU (**Hypothesis 7**, **H7**), (ii) *capacity* of CU has a positive impact on *demand* of SU (**Hypothesis 8**, **H8**), and (iii) *wait time* of CU has a positive impact on *wait time* of SU (**Hypothesis 9**, **H9**).

**Figure 3:**
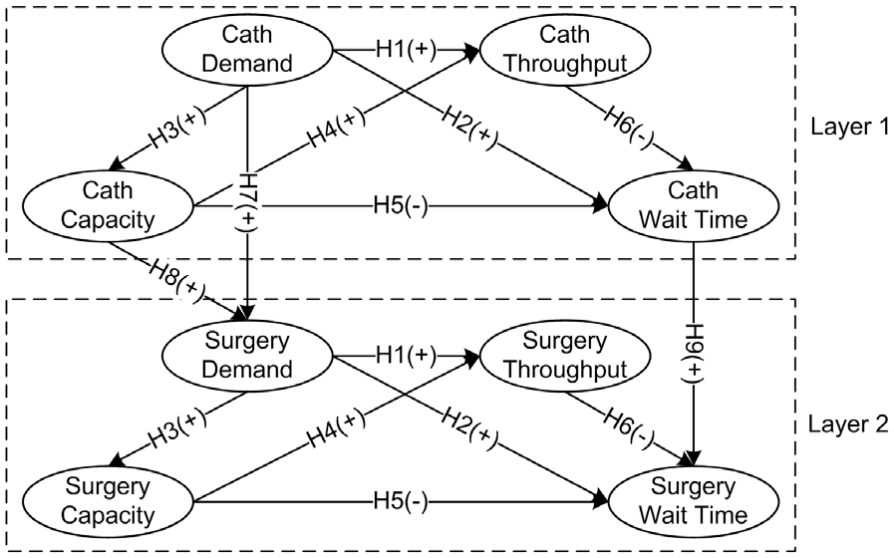
An illustration of a two-layer wait time model. (Cath: the abbreviation of catheterization; Surgery: the shorter form of cardiac surgery; H1-H9: the research hypotheses; +/−: a positive or a negative relationship between the variables towards the arrow.)

Based on the literature review, we postulate a two-layer wait time model (Figure 3) to represent the hypothesized within-unit and cross-unit wait time relationships. In this model, the relationships of four characteristics within the CU and the SU are illustrated in Layer 1 and Layer 2. Cross-unit wait time relationships are represented via the effects between the two layers.

### Cardiac Care Statistic Data

The data used in this study mainly comes from two data sources in Ontario, Canada. The first one is the Cardiac Care Network of Ontario (CCN, http://www.ccn.on.ca/), a network of 18 member hospitals providing cardiac services in Ontario. Since 2004, CCN reports the wait time facts quarterly for selected cardiac procedures (i.e., catheterization, cardiac surgery, and percutaneous coronary intervention) in member hospitals across Ontario. The reported data includes the number of completed cases in a month, the average number of patients waiting at the end of a month, and the monthly average median wait time. In this study, we are particularly interested in the units of catheterization and cardiac surgery, because a regional priority rating score system has been established for these two units (but not other units) in Ontario [32]–[33]. CCN thus provides more detailed statistics for CU and SU than for other units. Table 1 shows the major information provided by the CCN data. From Table 1, we can observe the variability of the throughput and the wait time for a specific unit.

**Table 1:** Cardiac Care Network of Ontario cardiac surgery statistics (January 2008–March 2008).

Hospital	C	UM(d)	SM(d)	EM(d)	W
Hamilton HSC	127	1	6	12	69
H 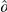 spital R*é*gional de Sudbury	36	7	6	19	21
Kingston General Hospital	47	3	15	20	30
London HSC	115	2	5	17	33
Southlake Regional HC	75	5	7	28	42
St. Mary's General Hospital	61	3	5	9	24
St. Michael's Hospital	89	5	6	15	26
Sunnybrook HSC	56	3	4	16	22
Trillium HC, Mississauga	79	2	4	9	22
University Health Network	129	2	6	13	135
University of Ottawa Heart Institute	98	6	21	52	100

C: the number of completed cases; UM: the median wait time of urgent patients; SM: the median wait time of semi-urgent patients; EM: the median wait time of elective patients; W: the number of waiting at the end of a month; d: the abbreviation of days. This table is drawn based on the CCN data (http://www.ccn.on.ca/pdfs/st-sur-2008-01-03.pdf).

We propose an equation (Equation 1) to calculate the monthly average number of arrivals from the existing statistic data, so that the demands of CU and SU can be estimated successfully.
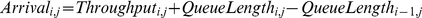
(1)where, 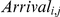 is the monthly average number of arrivals in quarter 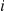 of unit 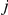, 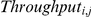 is the monthly average number of patients who have received treatment in quarter 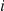 of unit 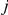, and 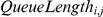 is the average number of patients waiting at the end of a month in quarter 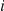 of unit 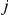. The second data source is the Ontario Physician Human Resources Data Center (OPHRDC, https://www.ophrdc.org/Home.aspx), a definitive source for information on physician usage in Ontario. It provides data about physicians in Ontario by specialties (e.g., cardiac surgery, diagnostic radiology) annually. In this study, the capacity of SU is exactly measured by the number of physicians specialized in cardiac surgery. The capacity of CU is approximately measured by the number of physicians operating diagnostic radiology, because catheterization is one of the tests utilizing radiology, and information about the physicians operating catheterization is unavailable. However, since the OPHRDC data is organized by Local Health Integration Networks (LHINs, not-for-profit corporations based on geographic regions to determine the community's health service needs and priorities), not by hospitals, it needs to be processed so as to align with the CCN data. Table 2 shows the CCN member hospitals and the corresponding LHINs. From this table, we can see direct correspondences between the LHINs and CCN Member Hospitals, except the LHINs of Toronto Central (TC) and North East (NE), which have more than one CCN hospital. To facilitate data analysis, the two LHINs' data should be decomposed to generate data for related hospitals.

**Table 2:** The relationship between CCN member hospitals and the LHINs.

LHIN	CCN Member Hospitals
South West	London Health Sciences Centre
Waterloo Wellington	St. Mary's General Hospital
Hamilton Niagara Haldimand Brant	Hamilton Health Sciences
Mississauga Halton	Trillium Health Network
Toronto Central	Toronto East General Hospitals*
	St. Michael's Hospital
	University Health Network
	Sunnybrook Health Sciences Centre
Central	Southlake Regional Health Centre
South East	Kingston General Hospital
Champlain	University of Ottawa Heart Institute
North East	Sault Area Hospital*
	H 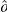 spital R*é*gional de Sudbury Regional Hospital

*: the hospital not providing the cardiac surgery procedure. This table is drawn based on the CCN information (http://www.ccn.on.ca/content.php?menuID=14&subMenuID=21&subMenu2ID=14).

The main idea behind data decomposition is to utilize hospitals' physician ratio (calculated from the number of specific physicians in a hospital to the total number of the specific physicians in the corresponding LHIN in year of 2010) in TC and NE to compute the number of physicians for relevant hospitals from 2005 to 2008. The physician ratios for CU and SU in each hospital in TC and NE can be obtained from the website of The College of Physicians and Surgeons of Ontario (CPSO, http://www.cpso.on.ca/), the governing body for medical doctors in Ontario. Then, after observing the OPHRDC data, we found that in TC and NE, the changes in CU ranged from 0 to 9 physicians per LHIN year to year (the total average number of catheterization physicians per hospital in the two LHINs was 60); and the changes in SU ranged from 0 to 1 physician per LHIN year to year (the total average number of cardiac surgery physicians per hospital in the two LHINs was 7). Therefore, we can assume that the physician ratios in TC and NE are relatively stable, i.e., the physician ratios are the same in each year since 2005. So that the number of specific physicians in each hospital can be calculated successfully by the specific physician ratio of each hospital multiplied by the number of the specific physicians in the corresponding LHIN each year.

By integrating and processing the two sets of data as discussed above, we obtain comprehensive information about the 11 hospitals (enumerated in Table 1) that provide catheterization and cardiac surgery. Table 3 outlines the characteristics of the two units and their measurements with the data summary. Specifically, we focus on the data from 2005 to 2008 (15 quarters in total), because the year of 2004 is the end of the first six-year cardiac expansion plan [7] and the start of the second ten-year cardiac improvement plan [25] [65]. In total, there are 165 data points for CU and SU (one hospital one quarter is regarded as a data point). In the next subsection, we will describe the statistical analysis methods used to investigate within-unit and cross-unit wait time relationships.

**Table 3:** A summary of the secondary data used in this study.

Characteristics	Measurements	CU	SU
Demand	Monthly average number of arrivals in a quarter	340	82
Capacity	Number of physicians, yearly	60	7
Throughput	Monthly average number of completed patients	346	83
Wait time	Median wait time of U/S/E patients	1/10/15	3/6/19
	Average number of waiting at the end of a month	101	58

CU: Catheterization unit; SU: Cardiac surgery unit; U: the urgent category; S: the semi-urgent category; E: the elective category.

### Statistical Analysis

In this study, we employ the structural equation modeling (SEM) to test the proposed two-layer wait time model (Figure 3) as well as the related hypotheses. The SEM is a second generation data analysis technique [66] for estimating complex relationships among multiple constructs [52]. The SEM and traditional statistic methods (e.g., regression, ANOVA, LOGIT) differ in important ways [54]: whereas traditional statistic methods can only test pairwise relationships between observed variables, the SEM can construct latent variables (abstract concepts that cannot be measured directly) and assess complex (e.g., hierarchical, recursive) causal paths among such variables. Therefore, the SEM technique has been increasingly used in social science, behavioral science and management science, for modeling complex and multivariate relationships [53] [67] [68] [69].

There are two classes of SEM: Partial Least Squares (PLS)-based SEM and covariance based SEM [54]. In this study (which is exploratory rather than confirmatory), the PLS-based SEM is employed because it is more suitable for theory building (i.e., allowing both confirmatory and exploratory modeling), whereas the covariance based SEM is more suitable for theory testing (i.e., more efficient in confirmatory modeling) [54].

In the data analysis process, the measurements for the *wait time* are modeled as formative indicators [54] [70] rather than reflective ones [54] [70]. A formative model is used when a latent construct (i.e., factor, such as *demand*, *capacity*, *throughput*, and *wait time* in this study) is viewed as an “explanatory combination” [71, p.422] of its manifest variables (i.e., measurements) [72]. In contrast, in a reflective model, the latent construct is viewed as causing the manifest variables [71]. In this study, the manifest variables for *wait time* are not interchangeable or correlated with one another because they measure the *wait time* from different perspectives. Therefore, the latent variable *wait time* is the summation of its corresponding manifest variables. In other words, the measurement items of *wait time* would be formative of the construct of *wait time*.

In addition, we utilize the data of CU and SU in the same quarter to test the cross-unit relationships. Because the longest wait time for a patient in the CU is around one month, we can assume that the great majority of patients who need cardiac surgery will be transferred from the CU to the SU within the period of a quarter. In the next section, we will present the results from the PLS analysis.

## Results

In this section, we discuss the findings of data analysis from two aspects: (i) how do the characteristics impact one another within a unit; (ii) how do the characteristics of CU impact the characteristics of SU, and particularly on *wait time* of SU.

**Figure 4:**
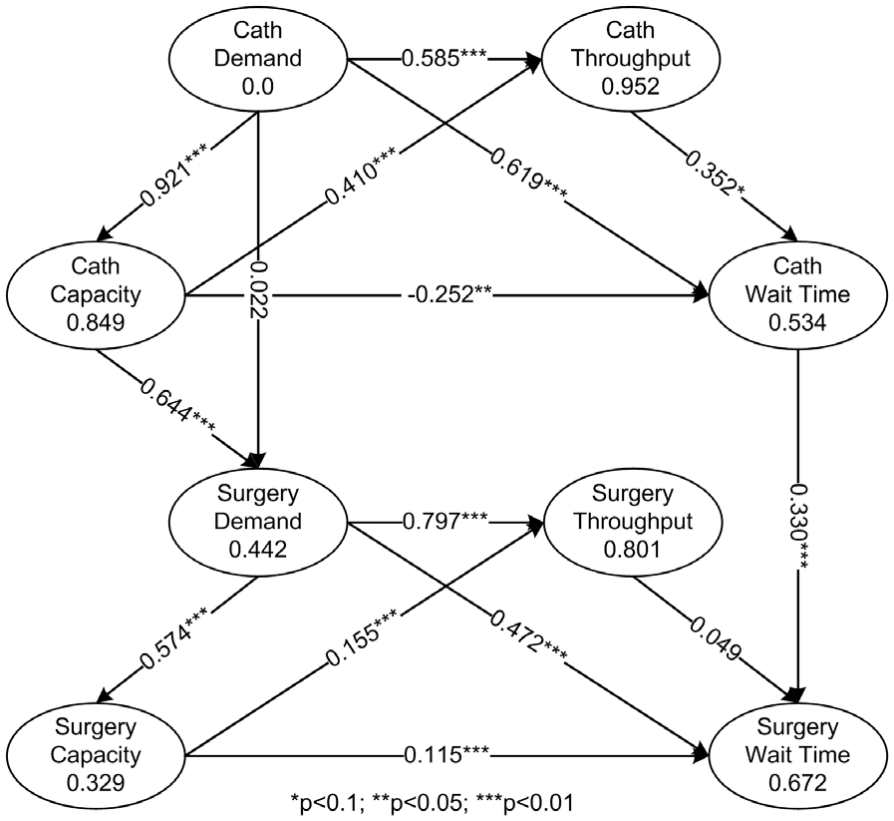
PLS test results based on a formative measurement model. (Cath: the abbreviation of catheterization; Surgery: the shorter form of cardiac surgery.)

In this study, the software SmartPLS (http://www.smartpls.de/) is utilized for path modeling and PLS-based data analysis. The results are shown in Figure 4.

### Within-Unit Relationships

As illustrated in Figure 4, in support of H1-H3, *demand* has a significant positive effect on *throughput*, *capacity*, and *wait time*, respectively. The path coefficients for the effect of *demand* on *throughput* for CU and SU are 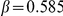 (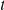 = 18.677, 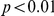) and 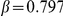 (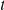 = 35.115, 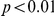), respectively. The path coefficients for the effect of *demand* on *capacity* are 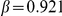 (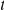 = 127.754, 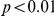) and 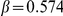 (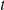 = 25.219, 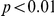) for CU and SU, respectively. The path coefficients for the effect of *demand* on *wait time* are 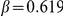 (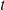 = 2.908, 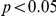) and 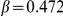 (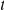 = 6.111, 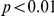) for CU and SU, respectively. These results confirm findings from prior research [21] [24] [35]–[37], providing further evidence that *demand* is an important predictor for *capacity*, *throughput* and *wait time* within a health care unit.

In support of H4, *capacity* has been found to have a significant positive impact on *throughput*. For CU, the path coefficient for the effect of *capacity* on *throughput* is 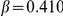 (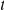 = 13.162, 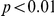). For SU, the path coefficient is 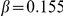 (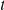 = 5.914, 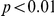). These results also confirm findings from prior research [38] [59], suggesting that improvement in capacity will lead to improved throughput within a unit.

Hypothesis H5 is only partially supported by our data. For CU, *capacity* has a significant negative impact on *wait time* (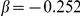, 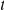 = 2.465, 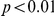), thus supporting H5. However, for SU, *capacity* has a significant positive impact on *wait time* (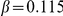, 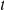 = 3.071, 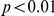). Thus H5 is not supported. This finding is different from that of prior research [35]–[36], which suggests that improvement in a unit's capacity can significantly shorten its patients' wait time.

The positive effect of *capacity* on *wait time* for SU can be explained by the view of Smethurst and Williams [39]–[40]. Their work figured out that the hospital waiting lists were “self-regulating” [39, p.652]. That means when capacity increases for meeting the demand, the demand also change in response, thus creating a demand that is even greater [39]–[40]. This is because a mass of “hidden” patients [39, p.653] (who have diseases but are not willing to go to hospitals) may be attracted to visit hospitals for believing be treated quicker. Hence, expanding the capacity in SU may help the wait time temporarily but, it will then increase, even get much longer than before because of more patients coming.

Hypothesis H6 is not supported by the data. Whereas *throughput* has a significant positive impact on *wait time* (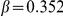, 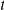 = 1.659, 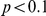) for CU, the effect of *throughput* on *wait time* is negligible for SU (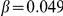, 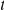 = 0.593, 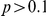). This finding suggests that *throughput* and *wait time* have similar changing patterns in CU (although not in SU), which is contrary to the expectation that the improvement of throughput results in the improvement of wait time.

A possible explanation for the positive relationship between *throughput* and *wait time* in CU can be found if considering the queue jumping behavior of urgent patients. Queue jumping means that urgent patients can skip the queue and jump to any position on a waiting list because of their treatment priority [73]. If more urgent patients arrive, units would like delay the treatment for the semi-urgent and elective patients in order to serve high priority patients in time, indirectly making these non-urgent patients wait longer. The overall wait time for the unit may also be increased as a result. In addition, the reason for the absence of any significant relationship between *throughput* and *wait time* in SU could be that SU has much fewer urgent patients than CU does. For instance, in the fiscal year of 2004, the percentage of urgent patients for CU in Ontario is 49% (out of a total of 52628 patients), while the percentage of urgent patients for SU is only 23% (out of a total of 7825 patients in total) [25]. This finding implies that in some cases, *throughput* and *wait time* may not be directly related to reflect the quality of a unit's performance.

### Cross-Unit Relationships

As show in Figure 4, H7 is not supported by our data (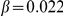, 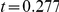, 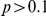). *Demand* of CU does not have a significant impact on *demand* of SU. While in support of H8, *capacity* of CU has a significant positive impact on *demand* of SU (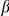 = 0.644, 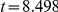, 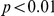).

The two findings can explain the formation of the “funnel and filter” effect [47, p.163] between the CU and the SU. Findings denote that on one hand, more arrivals in the CU usually lengthen the waiting list, but do not affect the throughput proceeding to the SU heavily. This may be because the CU always has a waiting list in reality (observed from the historical data published by CCN). On the other hand, to a large extent, the capacity of CU determines the actual numbers and the throughput for patients proceeding into the SU, so that the “funnel and filter” [47, p.163] is formed.

In support of H9, the results of our analysis reveal that *wait time* of CU has a significant positive impact on *wait time* of SU (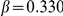, 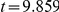, 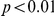). It provides strong evidence that *wait time* of CU is an important predictor for *wait time* of SU. A possible explanation for such an effect is delay cascade [74]. Unnikrishnan et al. [74] simulated and observed that delays would cascade in an emergency department (ED) network (all the EDs in different hospitals were networked by the transfer paths of ambulances). In other words, delays in an ED will result in wait time increasing in other EDs nearby. The cardiac care has a similar unit network (Figure 1) in a hospital. Therefore, delays in one unit may spread to other related units in the unit network, forming the direct cross-unit wait time relationship as a result.

Table 4 summarizes the hypotheses testing results. Besides, an examination of our results (Figure 4) reveals both direct and indirect causal paths from characteristics of CU to *wait time* of SU. In addition to a direct causal link from *wait time* of CU to *wait time* of SU, *demand* of CU and *capacity* of CU also have indirect effect on *wait time* of SU. In other words, *wait time* of SU may be influenced by the CU via the following causal paths: (i) *wait time* of CU 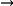 *wait time* of SU; (ii) *demand* of CU 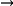 *capacity* of CU 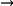 *demand* of SU 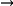 *wait time* of SU; (iii) *demand* of CU 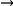 *capacity* of CU 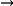 *demand* of SU 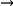 *capacity* of SU 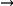 *wait time* of SU. *Demand* of CU appears to be the most essential driving force for the wait time dynamics in the CU as well as in the SU.

**Table 4:** A summary of hypotheses testing results.

Hypotheses	Supported?
H1-H4, H8, H9	Fully supported
H5	Partially supported
H6, H7	Not supported

## Discussion

In this study, we have examined whether and how characteristics of a preceding unit can affect the *wait time* of the cardiac surgery unit. Different from prior research, this study employs the structure equation modeling approach to assessing such cross-unit wait time relationships from the secondary data published in Ontario, Canada. The results of our analysis have validated the proposed two-layer wait time model, thus providing empirical support to the hypothesized relationships among four characteristics (i.e., *demand*, *capacity*, *throughput*, and *wait time*) both within a unit and across units.

The key findings in this study are as follows. First and foremost, our results show that *wait time* of CU has a direct positive impact on *wait time* of SU. This is a novel result, as prior research has seldom examined the influence of one unit's *wait time* on *wait time* of a subsequent unit on the patient flow process. A possible explanation for such effect is delay cascade in the cardiac care unit network (Figure 1), proposed by Unnikrishnan et al. [74].

In addition, the results of our analysis provide empirical evidence for previous findings that: (i) within a unit, *demand* has a positive effect on *capacity*, *throughput*, and *wait time*; (ii) within a unit, *capacity* has a positive effect on *throughput*; (iii) across units, the *demand* of one unit will be positively influenced by the *capacity* of the preceding unit.

We have also obtained some surprising findings: (i) the relationship between *capacity* and *wait time* differs in units with different profiles (e.g., different patient proportion in each urgency category); (ii) *throughput* has a positive effect on *wait time* within a unit; (iii) there exist direct and indirect wait time relationships between temporally-related units; (iv) *demand* of CU is an essential predictor for the other characteristics of CU and SU.

However, there may be other factors affecting a unit's performance in addition to *demand*, *capacity*, and cross-unit relationships. For example, the patient *risk* profile (i.e., the value of predicted operative mortality) has been identified as a factor that may affect the triage or referral patterns and the allocation of resources [75]. Although the exact effects of patient risk profiles on a health care system's performance (*wait time* in particular) are still unclear, it is desirable to explore their relationships in order to gain some insights in this regard by means of incorporating the information of patient risk into our two-layer wait time model.

**Figure 5:**
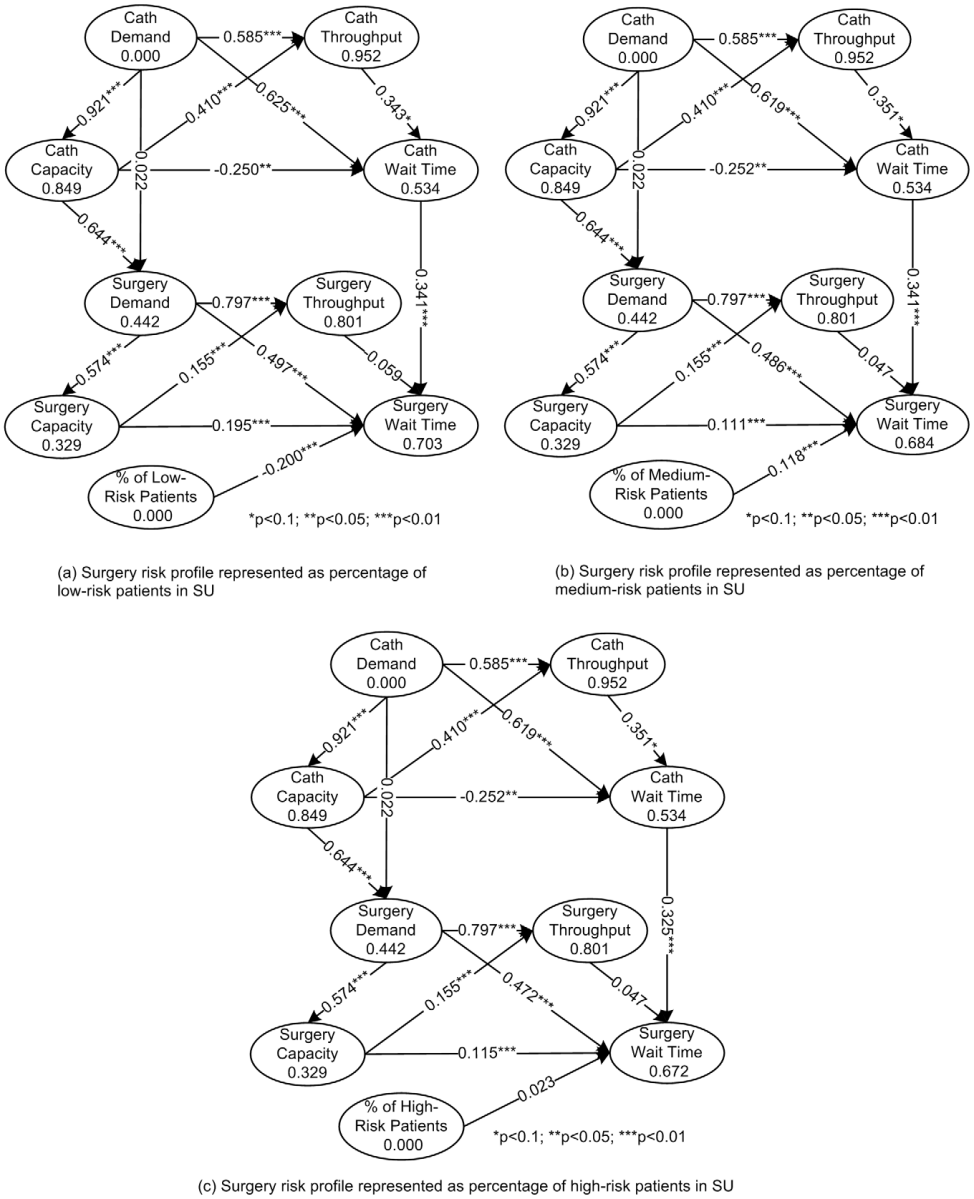
PLS test results for extended two-layer wait time model with risk profiles in SU. (Cath: the abbreviation of catheterization; Surgery: the shorter form of cardiac surgery.)

There are various methods for calculating the value of risk for patients undergoing catheterization (e.g., SYNTAX, http://www.syntaxscore.com/) and cardiac surgery (e.g., EuroSCORE, http://www.euroscore.org/, and Higgins Score [76]) based on several risk factors. For example, the surgical risk factors for isolated coronary artery bypass graft (CABG) surgery include age, sex, precious CABG, left ventricular function, and coronary anatomy, etc. [51] [77]. The Institute for Clinical Evaluative Science of Ontario has published data on the distribution of risk profiles in isolated CABG (i.e., the major type of cardiac surgery) in years of 2005 and 2006, in the Ontario hospitals [51]. Thus, by utilizing this published risk profile data (represented as the percentage of low-, medium-, and high-risk patients for catheterization in a hospital), we have further investigated the relationship between *risk* profiles and *wait time*. In doing so, the missing data of each hospital's risk profiles for the years of 2007 and 2008 is substituted by the mean value (a common method for handling missing data in statistical data analysis [78]–[79]) of its available risk data [51]. By integrating our original cardiac care data with the riskprofile data, we have conductd an additional PLS analysis to test the extended two-layer wait time model, with risk profiles added as an extra predictor of wait time in SU (see Figure 5).

The results of the analysis (Figure 5) reveal that the pattern of within- and cross-unit relationships (i.e., hypotheses H1-H9) among characteristics (i.e., *demand*, *capacity*, *throughput*, and *wait time* in CU and SU) remain unchanged. In addition, *risk* profiles, when represented differently (i.e., as percentage of *low-risk* patients, percentage of *medium-risk* patients, or percentage of *high-risk* patients), can have differential effects on *wait time* in SU.

More specifically, the percentage of *low-risk* patients has a significant negative effect on *wait time* (see Figure 5(a)). The exact explanation for this finding is still unclear as almost no prior work has addressed this issue to our best knowledge. However, it may be intuitively understood that the treatment process of low-risk patients is relatively easier than higher-risk patients, and hence, the length of stay (including the pre-operative, operating, and post-operative stay) of low-risk patients may be shorter than higher-risk patients. Therefore, if there are more low-risk patients in SU, the total wait time of this unit will be decreased.

Interestingly, the percentage of *medium-risk* patients has a significant positive impact on *wait time* (see Figure 5(b)). This may be due to the event of unexpected upgrading to more urgent categories (e.g., upgrading the medium-risk patients from semi-urgent category to urgent category) for patients proceeding to cardiac surgery [80]–[81]. The upgrading event may trigger the queue jumping behavior [73], which will hinder the normal treatment schedule and result in a longer wait time. This observation is consistent with the prior findings that proportionately more patients in the more urgent categories than in the less urgent categories may have wait times in excess of the maximum acceptable [82].

The percentage of *high-risk* patients does not have a significant effect on *wait time* (see Figure 5(c)), contrary to our expectation. Prior work indicates that high-risk patients tend to be assigned higher priorities in the triage process [80], and thus more high-risk patients may imply more urgent patients. Since urgent patients are more likely to undergo expedited surgery, this may delay the treatment for non-urgent patients, resulting in prolonged overall wait time [73]. Although, at the moment, we do not have a sound explanation for this unexpected lack of effect, the observed inconsistency between the effect of high-risk profile and that of medium-risk profile may be due to the actual methodology used to stratify patient risk profiles and priority categories, an issue that deserves further investigation.

Finally, the PLS-based SEM method proves to be an appropriate tool for assessing the hypothesized within-unit and cross-unit wait time relationships illustrated in our two-layer wait time model. With its capability of multivariate modeling and latent variable construction, the SEM approach enables us to validate the relationships among characteristics both within a unit and across two temporally-related units in this study.

It should be pointed out that there remain some limitations in this study. First, the CCN publishes only the monthly data averaged in a quarter. In order to avoid data overlapping, the time range of each data sample is thus set to 3 months. Secondly, since the number of physicians operating catheterization is unavailable, we have substituted it with the number of physicians specialized in diagnostic radiology. This substitution may not exactly reflect the true capacity of the catheterization. Also, we have used the current physician ratio obtained from CPSO to decompose the aggregated OPHRDC data from LHIN-based to hospital-based. Data produced by this conversion process may not be very accurate because the physician ratio may change from year to year. Moreover, we have used only one indicator for *demand*, *capacity*, and *throughput*, which may not capture all the dimensions of the relevant constructs. Nevertheless, this study represents a valuable attempt to use the SEM method to explore factors affecting wait time from a multi-unit perspective, based on secondary data. Our findings can also provide valuable insights to researchers and practitioners in other government dominated health care systems in their efforts to reduce wait time.
